# Parkinson's Disease and Mesenchymal Stem Cells: Potential for Cell-Based Therapy

**DOI:** 10.1155/2012/873706

**Published:** 2012-02-28

**Authors:** Masaaki Kitada, Mari Dezawa

**Affiliations:** Department of Stem Cell Biology and Histology, Tohoku University Graduate School of Medicine, Sendai 980-8575, Japan

## Abstract

Cell transplantation is a strategy with great potential for the treatment of Parkinson's disease, and many types of stem cells, including neural stem cells and embryonic stem cells, are considered candidates for transplantation therapy. Mesenchymal stem cells are a great therapeutic cell source because they are easy accessible and can be expanded from patients or donor mesenchymal tissues without posing serious ethical and technical problems. They have trophic effects for protecting damaged tissues as well as differentiation ability to generate a broad spectrum of cells, including dopamine neurons, which contribute to the replenishment of lost cells in Parkinson's disease. This paper focuses mainly on the potential of mesenchymal stem cells as a therapeutic cell source and discusses their potential clinical application in Parkinson's disease.

## 1. Introduction

In the central nervous system, where neurons become postmitotic after birth, little structural and functional regeneration occurs. Although intrinsic neural stem cells and progenitor cells proliferate and differentiate after damage, their contribution is insufficient for functional recovery [1]. Protective treatment effectively prevents the progressive loss of dying neuronal cells in the earlier stages of neural degeneration, but in advanced stages, transplantation of cells with neuronal properties is considered the ultimate solution for degenerative diseases. Parkinson's disease (PD) is a neurodegenerative disease caused by the loss of midbrain dopamine neurons with a subsequent decrease in striatal dopamine [2]. Pharmacologic treatment with the dopamine precursor L-DOPA is effective in the earlier stages, but reduced efficacy and the development of motor complications in the later stages require treatment alternatives such as dopamine neuron transplantation [3].

 Transplantation of embryonic mesencephalic cells or fetal dopamine cells into the striatum of PD patients was initiated in the 1980s [2]. Some studies have reported negative effects of human embryonic or fetal dopamine neuron transplantation, that is, clinical benefits were recognized in younger patients but not in older patients, and more than half of the transplanted patients developed dyskinesia that persisted after overnight withdrawal of dopaminergic medication [4, 5]. On the other hand, other groups have reported positive results of the transplantation of embryonic or fetal dopamine neurons. Grafts of embryonic dopaminergic neurons can survive and exert functional effects for up to several years after surgery in the brain of patients with PD [6, 7]. In addition, sufficient recovery with the integration and reinnervation of grafts is observed in positron emission tomography [8]. While these transplantation strategies raise hopes for reverting PD, there are limiting factors that preclude the therapeutic use of embryonic and fetal cells, such as ethical issues and obtainable cell numbers. Only small numbers of dopamine precursors can be collected from donor embryonic or fetal tissues. Therefore, there is a great need for generating large pools of dopamine neurons or precursors for transplantation.

 Stem cells have recently aroused a great deal of interest because of their potential to differentiate into dopamine neurons either by spontaneous differentiation or through certain induction protocols [3]. Neural stem cells (NSCs) and embryonic stem (ES) cells have been studied for more than decade. More recently, several intensive studies have focused on the use of mesenchymal stem cells (MSCs). In this paper, we describe the advantages and disadvantages of each stem cell type with regard to its potential use for PD treatment, focusing mainly on MSCs.

## 2. MSCs and Their Properties

**Figure 1:**
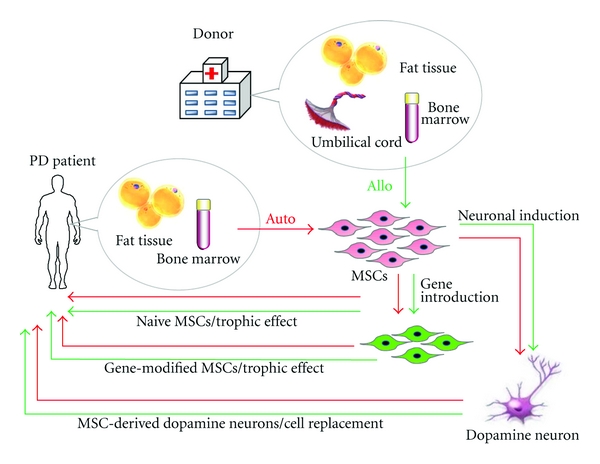
Strategy for MSC transplantation in PD patients. MSCs can be obtained from fat tissue or bone marrow aspirates of Parkinson's disease (PD) patients and are applicable for autocell transplantation. They can also be obtained from fat tissue, bone marrow aspirates, and umbilical cord of healthy donors for allocell transplantation. Naive MSCs can be directly transplanted into the striatum of PD patients, but this treatment exerts temporary trophic effects. Gene-introduced MSCs also have trophic effects for the replenishment of lost cells. MSCs are able to be induced into dopamine neurons that will contribute to the functional recovery of PD>.

 MSCs are adult stem cells that belong to the mesodermal lineage and are traditionally found in the bone marrow as bone marrow mesenchymal stem cells (BMSCs) [9]. MSCs can also be isolated from other mesenchymal tissues, such as umbilical cord, dermis, adipose tissue, and peripheral blood [10]. Morphologically, MSCs have long thin cell bodies with a large nucleus similar to fibroblasts. As with some other tissue stem cells, MSCs have a high capacity for self-renewal while maintaining multipotency [11]. Different from other stem cells such as ES cells and NSCs; however, MSCs can be obtained from patients (for autocell transplantation) as well as from healthy donors (for allo-transplantation) by using mesenchymal tissues such as fat, bone marrow, and umbilical cord (Figure 1). Therefore, MSCs are a realistic cell source for regenerative medicine.

 Among the many kinds of MSCs, BMSCs are the most well studied. BMSCs can be cultivated from bone marrow aspirates as plastic adherent cells *in vitro *[11]. The great benefit of BMSCs is that they are easily accessible through aspiration of the patient's bone marrow, so that the use of BMSCs avoids ethical issues, facilitating their application both for auto- and allo-transplantation. BMSCs are also easily expanded on a large scale, which is very convenient for clinical use (e.g., 20 to 100 mL of bone marrow aspirate provides 10^7^ BMSCs within several weeks) [12].

 For cell-based therapy, MSCs have two major effects: a trophic effect that is mediated by the various types of trophic factors and cytokines produced by MSCs [13] and differentiation to generate a broad spectrum of cells for the replenishment of lost cells [14]. MSCs normally provide trophic factors to support hematopoietic stem cells in the bone marrow, thus their trophic effect is part of their normal function. MSCs are multipotent stem cells that are known to differentiate into osteocytes, chondrocytes, and adipocytes [11]. These differentiations are within the same mesodermal lineage, but recent reports demonstrated that MSCs show unorthodox differentiation into ectodermal and endodermal cells [15–19]. These findings stimulated the advancement of regenerative medicine aimed at the generation of desired cells from MSCs. To date, various cell types, such as mesodermal lineage cells (e.g., bone, cartilage, adipocytes, skeletal muscles, and cardiomyocytes), as well as endodermal lineage cells (e.g., airway epithelial cells, hepatocytes, and insulin-producing cells) and ectodermal lineage cells (e.g., neuronal cells and epidermal cells) have been induced from MSCs *in vitro* by the use of cytokines, trophic factors or gene introduction [15–22].

 Adult stem cells typically generate the cell types of the tissue in which they reside, and thus the range of their differentiation capabilities is considered limited. For example, hematopoietic stem cells generate blood cells, and NSCs generate neurons and glial cells [23, 24]. MSCs differ from these typical somatic stem cells because, as stated previously, they differentiate not only into the same mesodermal-lineage cells of bone, cartilage, and adipocytes, but also into other lineages of ectodermal and endodermal cells. As MSCs can generate cells representative of all three germ layers, it has been debated whether MSCs are pluripotent cells. Recently, pluripotent stem cells named multilineage-differentiating stress enduring (Muse) cells were found among adult human mesenchymal stem cells (BMSCs and skin fibroblasts) as well as in mesenchymal tissues (bone marrow and dermis) [25]. Muse cells are capable of self-renewal and of differentiating into cells representative of all three germ layers from a single cell, which may partly explain the broad spectrum of differentiation observed in MSCs [25].

## 3. MSCs and Their Differentiation Ability

The possibility of MSC plasticity and “transdifferentiation” was initially described following *in vivo *experiments in which transplanted donor bone marrow-derived cells differentiated into glial cells in the recipient brain [26]. While some studies suggested that MSCs are plastic based on their expression of cell-specific markers, the functions of the transdifferentiated cells were not clearly demonstrated in other cases. Moreover, questions have been raised regarding the interpretation of “transdifferentiation” of infused cells into neuronal lineage cells because some investigators have suggested that the transdifferentiation observed was rather a result of fusion between infused bone marrow cells and the host brain cells [27, 28]. Despite this uncertainty, accumulating evidence supports the broad differentiation of MSCs both *in vivo* and *in vitro*. Based on the frequency and ratio of MSCs integrated and differentiated into the host tissue, fusion alone cannot explain all of the phenomena observed after MSC infusion. Furthermore, experiments using a Cre-lox system clearly demonstrated that MSCs can transdifferentiate into epithelial cells *in vivo* without fusion [29]. *In vitro* differentiation of MSCs provides further evidence for MSC transdifferentiation because there are no preexisting differentiated cells to be fused at the beginning of induction under culture conditions.

## 4. BMSCs

 There have been many attempts to infuse BMSCs into a PD model aimed at ameliorating PD symptoms. As mentioned previously, BMSCs have trophic effects that are mediated by the various types of trophic factors and cytokines they produce. Therefore, naive adult BMSCs engrafted to the striatum induce partial but not drastic recovery of the dopamine pathway in a rat model of PD (Figure 1) [30–34]. Findings from a human pilot study of autologous naive BMSC transplantation performed in PD patients and followed for up to 36 months indicated a certain degree of amelioration of symptoms with no tumor formation [35]. While BMSCs have advantages over some other stem cells regarding their safety, easy accessibility, and trophic effects, naive BMSC transplantation has limitations for definitive care because most of the transplanted cells do not survive *in vivo* for a long time, and thus the trophic effects gradually decrease.

 In addition to naive BMSC transplantation, genetically modified BMSCs have been applied to the PD model (Figure 1). Cells genetically modified to produce L-DOPA or neurotrophic factors such as neurotrophins and glial cell line-derived neurotrophic factor (GDNF) are reported to be somewhat effective for the amelioration of PD symptoms [36–39].

 While naive BMSC transplantation is indeed a simple and accessible method for providing trophic effects, dopamine neurons would be a rational ultimate solution to PD (Figure 1). Naive BMSCs, in general, do not differentiate spontaneously *in vivo* after transplantation. Even if they did differentiate, the ratio of differentiated cells would be extremely low [26]. For practical use, it would be more desirable to establish a specific system for inducing BMSCs to produce dopamine neurons prior to transplantation.

 There are several reports of the induction of dopamine neurons from BMSCs [40–42], but in these reports the effectiveness of the induced cells *in vivo* was not evaluated by transplanting them into a PD model. Another study reported that MSCs induced into immature neurons using basic fibroblast growth factor (bFGF), epidermal growth factor, platelet-derived growth factor, sonic hedgehog, FGF-8, GDNF, or the reagents butylated hydroxyanisole and dibutyryl cAMP were transplanted into a PD model, but these immature neurons did not effectively ameliorate the PD symptoms [43, 44]. In this manner, growth factor-based methods allow MSC differentiation toward immature neuronal-like cells, but are not efficient in PD models. On the other hand, when MSCs were induced into fully functional dopamine neurons and then transplanted into a PD model, they were clearly effective, as described in the next section [17].

## 5. Induction of Functional Dopamine Neurons from BMSCs

**Figure 2:**
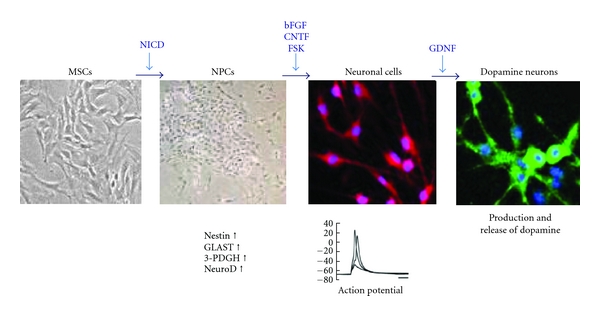
Induction of dopamine neurons from MSCs. After NICD introduction, MSCs become similar to NPCs, expressing the NPC markers nestin, GLAST, 3-PDGH, and neuroD. After cytokine stimulation (bFGF, CNTF and forskolin (FSK)), cells become postmitotic neurons expressing neuronal markers such as neurofilament, Tuj-1, and MAP-2. The administration of GDNF induces neurons to become dopamine neurons (TH), which are useful in the Parkinson's disease model. Pictures from J Clin Invest 113 (2004) 1701–1710 and J Cereb Blood Flow Metab 29 (2009) 1409–1420 [17, 46].

 A system to specifically induce dopamine neurons from BMSCs was reported (Figure 2) [17]. This system first generates postmitotic functional neuronal cells with a very high efficiency without contamination by glial cells. The resulting neuronal cells are then further induced into dopamine neurons. The induction is achieved by lipofection of a plasmid containing a Notch1 intracellular domain (NICD) and G418 selection, followed by the administration of a specific combination of trophic factors and cytokines [17, 45]. 

 Naive BMSCs initially show little expression of the glutamate transporter GLAST, 3-PDGH, and nestin, which are markers for neuronal progenitor cells (NPCs), but BMSCs express these markers substantially after introduction of NICD (Figure 2). Based on a luciferase promoter assay, promoter activities of 3-PDGH, which are reported to be high in radial glia and neuroepithelial cells, as well as those of the neuronal marker NeuroD, are significantly increased (up to 10 times) in BMSCs after NICD introduction. These findings suggest that the introduction of NICD into the cells induces BMSCs to acquire the characteristics of NPCs (Figure 2) [17, 46].

 When NICD-introduced BMSCs are expanded and then stimulated with trophic factors (bFGF, forskolin, and ciliary neurotrophic factor (CNTF)) for several days, approximately 96% of the cells extend neurites and differentiate into postmitotic neuronal cells. These cells are positive for the neuronal markers MAP-2ab, neurofilament, and Tuj1, and most importantly, action potentials were recorded in the cells in a patch clamp experiment, suggesting that these induced cells are functional neuronal cells (Figure 2) [17, 47].

 At this stage, cells positive for tyrosine hydroxylase (TH), a marker for dopamine neurons, accounted for ratios of only approximately 4%. After GDNF stimulation, the cells positive for TH substantially increased up to ~60% (Figure 2). Furthermore, other dopamine markers, Nurr-1, Lmx1b, En1, and Ptx3, were elevated in these TH-positive cells [17]. The dopamine release upon depolarization *in vitro* measured by high-performance liquid chromatography indicated that the induced cells released dopamine into the culture media in response to high K+-depolarizing stimuli. These findings indicate that functional dopamine neurons can be efficiently induced from BMSCs (Figure 2) [17].

 The adaptability of BMSCs to an* in vitro* environment and their proliferative activity differ among species. In general, human and rat BMSCs can stably proliferate *in vitro* while those of monkeys and mice are vulnerable to manipulation, often resulting in unsuccessful NICD gene introduction by lipofection due to the cytotoxicity of lipofection. Cell damage during the induction procedure is a barrier to realizing cell-based therapy, and thus, gene introduction with higher efficiency and safety is strongly needed for practical use. Spermine/pullulan-mediated reverse transfection is an effective method for introducing plasmid genes, even into vulnerable cells, with high efficiency and low cytotoxicity. In fact, introduced NICD genes are successfully transcribed and expressed as protein in the cytoplasm of monkey and mice, as well as in human BMSCs, with extremely low levels of cytotoxicity [48]. This system is also effective for inducing dopamine neurons from monkey and mice BMSCs. NICD introduction into BMSCs using spermine/pullulan-mediated reverse transfection followed by cytokine administration successfully induces neuronal cells that show dopamine release in high-performance liquid chromatography [48]. Thus, the spermine/pullulan-mediated reverse transfection is an ideal alternative method to induce dopamine neurons from BMSCs of a wide range of species.

## 6. Transplantation of BMSC-Derived Dopamine Neurons into PD Models

 Induced dopamine neurons (1 × 10^5^ cells) from either rodent or human (under the control of immunosuppressant) BMSCs were transplanted into the striatum of a PD model rat induced by 6-hydroxydopamine (6-OHDA) [17]. Unilateral administration of 6-OHDA into the medial forebrain bundle selectively destroys dopamine neurons in the substantia nigra, leading to quantifiable changes in rotational behavior and providing a useful and commonly used model of PD. Model rats receiving a transplantation demonstrated a substantial decrease in apomorphine-induced rotation behavior, and nonpharmacologic behavior tests, such as adjusting step and paw-reaching tests, also demonstrated significant improvements in both rodent and human induced cell transplantation. Grafted dopamine neurons migrated and extended beyond the injected site, and approximately 30% of the cells remained in the striatum 10 weeks after transplantation. The grafted striatum showed the migration of GFP-positive transplanted cells that expressed neurofilament, TH, and DAT. Brain slice culture experiments demonstrated the production of dopamine in the transplanted brains. No tumor formation was observed in the brain, demonstrating that dopamine neurons induced from BMSCs do not have the ability to form tumors [17].

 In summary, introduction of NICD followed by bFGF, CNTF, forskolin, and GDNF administration can efficiently induce functional dopamine neurons that lead to functional recovery after transplantation in a rodent model of PD.

 Notch signaling inhibits neuronal differentiation and promotes glial differentiation during development [49]. Although the above discussed induction system seems inconsistent with the well-known actions of Notch signaling, it is presumed that cell susceptibility to Notch signaling in MSCs is different from that of cells in the process of normal neuronal development. Distinct cellular responses to Notch signals; for example, the protein repertoire and active factors, might be quite different between conventional NPCs and BMSCs. In fact, neuronal basic helix-loop-helix factors (Mash1, Math1, and neurogenin1), together with the glial factors Hes1, Hes5, STAT1, and STAT3, are detected in naive BMSCs in reverse transcription-polymerase chain reaction analyses, while after NICD transfection, expression of STAT1 and STAT3 is downregulated and expression of Mash1, Math1, and neurogenin1, as well as Hes1 and Hes5, is retained in the BMSCs [17]. Although it is believed that the major intracellular effect of NICD introduction is the activation of Hes1 and Hes5, the introduction of either Hes1 or Hes5 to BMSCs, instead of NICD, does not induce NPC marker-positive cells. In contrast, administration of the Janus kinase (JAK)/STAT inhibitor WHI-P131, instead of NICD transfection, successfully produces NPC-like cells, which are partially induced to be MAP2-antibody-positive cells with neurite-like processes after additional trophic factor induction [17]. These facts suggest that the downregulation of STAT expression by NICD-transfection is closely related to the transformation of MSCs to NPC-like cells and that Hes activity is not involved in this process.

## 7. Other Kinds of MSCs and PD

 The umbilical cord and adipose tissues are other realistic sources of MSCs. Mesenchymal tissues of the umbilical cord, so-called Wharton's jelly, as well as fat tissues, contain an abundance of MSCs. These cells have an advantage over BMSCs in that the umbilical cord derives from postnatal tissue that is discarded after birth, and thus cell collection is not an invasive procedure for donors. Adipose tissue, which is easily obtained from liposuction, also contains large amounts of MSCs called adipose-derived stem cells (ADSCs). Because of the ability of umbilical cord mesenchymal stem cells (UC-MSCs) and ADSCs to differentiate into other cell types and to proliferate, these cells are considered to be a practical source for cell-based therapies.

 In ADSCs, Tuj-1-positive cells, but not fully differentiated dopamine neurons, induced, and transplanted into a PD model, demonstrated that these neuron-like cells are effective for treating PD to a certain degree after transplantation [50].

 As for UC-MSCs, transplantation of naive cells and cells genetically modified to produce VEGF were partly effective [51–53]. The potential of UC-MSCs to differentiate into neuronal cells does not differ from that of BMSCs, and dopamine neurons can be induced from UC-MSCs using neuron-conditioned medium, sonic hedgehog, and FGF-8. Those cells are also effective in PD models [54–56].

## 8. MSCs and Clinical Studies

 A human pilot study was performed using BMSCs. Autologous naive BMSCs were transplanted into PD patients and the patients were followed for up to 36 months. This clinical trial resulted in partial symptom amelioration without evidence of tumor formation or other side effects [35]. Clinical studies of MSCs have just begun, and there is a strong need to accumulate results regarding MSC transplantation and its efficacy.

## 9. NSCs

 NSCs are an attractive source for cell replacement therapy for PD because they have the ability to differentiate into neurons, astrocytes, and oligodendrocytes as well as into dopamine neurons [23, 57]. NSCs can be isolated from different regions of the fetal brain as well as from the ventricular wall and the hippocampal dentate gyrus in the adult brain so that they can be harvested both from fetal and adult central nervous system tissues [3]. As NSCs are able to self-renew, they can be maintained and expanded either as monolayers or as floating aggregates called neurospheres [3]. Repeated expansion of NSCs is, however, reported to decrease their potential to differentiate into a variety of neuron subtypes, including dopamine neurons. In particular, adult NSCs have lower ability for differentiation than fetal NSCs [3]. Limitations in the use of NSCs include ethical and histocompatibility concerns for fetal NSCs and a limited supply of adult NSCs.

 The first results of genetic manipulation of NSCs to generate dopamine neurons by overexpression of the transcription factor Nurr 1 were reported in 1999 [57]. Gene introduction of the combination of Nurr1/Ngn2 or Nurr1/Mash1 effectively induced dopamine neurons, but the induction efficiency was at most 1% so that these systems seem not to be directly clinically applicable for PD [58, 59].

## 10. ES Cells

 ES cells have attracted great attention as an alternative source for the generation of dopamine neurons because they can be continually expanded with high potential for differentiation. As they are pluripotent stem cells, they are able to form all three embryonic germ layer lineages following induced differentiation. Human ES cells were first derived in 1998 by Thomson [60], and since then many studies have focused on optimizing the differentiation of ES cells into dopamine neurons. Among them, systematic and efficient induction systems for dopamine neurons have been reported by several groups [61–64], and mouse ES cell-derived dopamine neurons have been shown to survive and function in a rat model of PD [65].

 The prospect that ES cells can produce a sufficient number of dopamine neurons for transplantation therapy is particularly appealing, both for clinical and industrial use. At the same time; however, their clinical application is limited because of their ability to form tumors and the ethical problems surrounding the use of using fertilized human eggs to establish the ES cell lines. In addition, autocell transplantation is unrealistic in the case of ES cells.

 In particular, several reports have raised an alert regarding the tumor-forming ability of ES cells. For example, engraftment of neurally selected ES cells to eyes exhibited no morphologic alterations by 2 and 4 weeks, whereas at 8 weeks, neoplasia formation was detected in 50% of the eyes in almost all layers of the eye, including the retina, vitreous, and choroid [66]. These neoplasias expressed the characteristics of the different germ layers, so they were considered to be teratomas. Even if ES cells seem fully differentiated into dopamine neurons *in vitro* before transplantation, they still carry the risk of tumorigenesis. For example, a rat PD model grafted with mouse ES cells predifferentiated into dopamine neurons developed severe teratomas [67, 68]. Thus, ES cells may provide treatment for degenerative disease in the future, but their unlimited self-renewal and proliferative potential pose the risk of tumor induction after engraftment, which is a difficult obstacle that must be overcome.

## 11. iPS Cells

Induced pluripotent stem (iPS) cells, whose properties are similar to those of ES cells, can be generated from adult human cells, such as dermal fibroblasts, by introducing genes such as Sox2, Oct3/4, Klf-4, and c-Myc [69]. They have thus attracted increasing attention as a new type of pluripotent stem cell without major ethical concerns. As is the case with ES cells, iPS cells can generate cells of all three germ layers and have unlimited proliferative activity, but their clinical application is limited by their tumorigenicity. iPS cells can be induced to form dopamine neurons, but the induction efficiency is generally lower than that of ES cells and the quality of the cells is not homogenous [69, 70]. One recent report suggested that iPS cells induced from mouse fibroblasts are able to integrate into the fetal brain, and can improve symptoms in a rat PD model, but similar to ES cells, they carry the risk of tumorigenesis [71].

## 12. Other Cells

 Retinal pigment epithelium is a recent potential candidate for cell therapy in PD because L-DOPA, which is produced during the metabolic pathway of melanin in retinal pigment epithelium, metabolizes to DOPA when taken up into the glial and neuronal cells. The produced DOPA affects PD so that if retinal pigment epithelia survive and integrate after transplantation, this strategy will be beneficial [72]. The system will not be applicable for autocell transplantation; however, so the cell sources remain a problem. In phase I of study, retinal pigment epithelia collected from cadaveric tissues were transplanted into PD patients, but no antiparkinsonian benefits have been recognized.

## 13. Perspectives

While ES cells and NSCs have great potential, MSCs provide strong possibilities for clinical application, because they are easily accessible cells with few ethical problems and can be efficiently expanded *in vitro* to achieve therapeutic scale. Importantly, MSCs are already widely used clinically to treat osteoarthritis and myocardial infarction, so they have an established record in clinical applications. Furthermore, they are easily obtained from patients or marrow banks, autologous transplantation, or transplantation with the same HLA subtype from a healthy donor, which may minimize the risks of rejection.

 Although transplantation of naive MSCs is effective for treating PD, this is mostly due to the trophic effects, which do not persist for a long period. In addition, cell transplantation via intravenous administration is known to occasionally cause pulmonary thrombosis when the cells are infused in a high concentration [73]. Therefore, from the perspective of cell based therapy, it is desirable to transplant functional dopamine neurons induced from MSCs directly into the striatum of PD patients by stereotaxic operation [17]. Because the dopamine neuron induction using NICD transfection involves plasmid introduction, further long-term studies are needed to ensure safety against tumor formation and efficacy of manipulated MSCs.

 MSCs are usually harvested as adherent cells from mesenchymal tissues and are thus a heterogeneous cell population [10, 25]. Any of these types of adherent cells could contaminate the MSC population, particularly in the initial step of culture. In subsequent subcultures, the cells seem to converge on general MSCs, and other cell types are left out, but still MSCs do not comprise a single homogeneous cell type. Therefore, the big picture of MSCs is not yet clarified, and in fact, a specific molecular marker that is exclusively expressed by MSCs has yet to be found. For these reasons, the entity of differentiation into dopamine neurons remains an enigma. Indeed, MSCs show a trilineage differentiation, but the differentiation ratio is usually not very high and thus a subpopulation of MSCs seems to be related to the differentiation. The pluripotent stem cells, Muse cells, were identified in human mesenchymal tissues and cells [25]. If the cells responsible for the differentiation of dopamine neurons are clarified, the potential of MSCs for application to PD will be greatly advanced.
